# A dataset of geotechnical parameters based on international literature to characterise lithotypes in Italy

**DOI:** 10.1038/s41597-024-04095-1

**Published:** 2024-12-18

**Authors:** Nunzia Monte, Francesco Bucci, Federica Angela Mevoli, Michele Santangelo, Paola Reichenbach, Lucio Di Matteo, Ivan Marchesini

**Affiliations:** 1 CNR IRPI, via della Madonna Alta 126, 06128 Perugia, Italy; 2 Dipartimento di Fisica e Geologia, Università degli Studi di Perugia, Via A. Pascoli snc, 06123 Perugia, Italy

**Keywords:** Geology, Natural hazards

## Abstract

Geological and lithological maps provide essential spatial data for various environmental assessments and studies. However, these maps lack detailed quantitative information on the geotechnical characteristics of rocks and soils, which limits their use for modelling purposes. This study addresses this gap by compiling a comprehensive database of over 2300 geotechnical parameter records searching the international literature. Focusing on cohesion, friction angle, and porosity, we analyse their distributions across different lithotypes, emphasising their significance in slope stability modelling. For the Italian territory, the collected information was used to associate geotechnical parameters to the lithological classes as identified by Bucci *et al. 2022*. These types of reclassified maps may provide researchers and stakeholders with a comprehensive dataset useful for slope stability assessment and land management at small scale. Descriptive statistics and validation from grey literature underscore the dataset’s utility in enhancing geotechnical characterizations and supporting geological hazard assessments.

## Background

In the past decades, geological^1–3^ and lithological maps^4–7^ have been produced and published for entire national territories, at various scales by research institutes, technical organisations, and official institutions. Geological maps illustrate the spatial distribution of different formations and the locations of structures such as faults and folds^8–12^ whereas lithological maps encode information on the geochemical, mineralogical, and physical properties of the rocks^6,8,13,14^.

Geological and lithological maps are relevant for several different evaluations and purposes including: (i) environmental assessment (e.g., understanding the potential for soil erosion^15^); (ii) slope stability analysis^16^; (iii) production of soil maps^17,18^; (iv) land use planning (e.g., determining suitable locations for infrastructure development, based on the geological stability of the area^19,20^); (v) evaluation of geo-hydrological hazards such as floods^21^ and landslides^22^; and (vi) studies on climate change (e.g., analyses of past geological records to understand historical climate changes, or study of the impact of climate change on geological processes^23^). Unfortunately, geological and lithological maps lack quantitative information on the geotechnical characteristics of rocks and soils, which limits their use for modelling purposes. Geotechnical characteristics refer to the mechanical, physical, and hydraulic properties of geo-materials and play a key role in evaluating the behaviour of rocks and soil in various contexts. Values of the geotechnical properties can be measured, estimated or evaluated and used in several types of site specific and regional models and analyses. The number and type of parameters is often related to the context and the specific requirements of the analysis. Parameters include for example, soil shear strength, hydraulic conductivity, compressibility, cohesion, and internal friction angle. In this paper, we focus on effective cohesion, effective friction angle, and porosity, which are essential for slope stability modelling.

Cohesion represents the non-frictional part of the shear strength. It mainly depends on cementation, adhesion, packing, electrostatic and electromagnetic attraction^24–29^. Friction angle is an indicator of the resistance to shear forces related to particle size distribution, angularity and particle interlocking, in addition to mineralogy^29–31^. Porosity is the measure of the amount of voids within a rock formation or soil^32^. In the geotechnical context, porosity is fundamental for understanding the hydrogeological characteristics of the soil, influencing water content.

Reliable data for characterising the physical, mechanical, and hydraulic properties of soils and rocks can be obtained with different types of investigations both in the laboratory and in the field. In addition to laboratory and field tests, this information can be obtained through estimates or empirical relationships, as proposed by^33^, who derived information on porosity using uniaxial tests. The selection of specific tests depends on the project requirements and the type of materials. Fine-grained soils (e.g., clayey or silty geo-materials) can be collected as undisturbed samples during field surveys. For these types of materials, strength parameters are typically determined through laboratory tests, such as direct shear^34^ and triaxial compression tests^35,36^. Conversely, *in-situ* testing is preferred to derive the strength parameters of coarse-grained soils (e.g., sandy or gravelly geo-materials), since the collection of undisturbed samples is challenging. This is typically achieved through tests such as the Standard Penetration Test^37^. *In-situ* Cone Penetration Tests^38^ are also suitable for fine-grained soils. The geo-mechanical characterization of rock masses usually involves laboratory tests such as the Uniaxial Compression Test^39^ for deriving compressive strength and direct or indirect Tensile Tests^40,41^ for determining tensile strength. Porosity is usually determined in the laboratory through Mercury intrusion porosimeter^42^, but it can also be estimated from dry density^43^ and P-wave velocity^44^.

Methods based on laboratory and field tests provide information and values for single points (in the three-dimensional space) and it is very difficult to get a representation of their continuous spatial distribution, which is rarely derived and only in limited study areas (e.g.^45–47^). Distributed data is a major challenge that may depend on multiple factors, including human and economic resources.

In this paper, we describe the results of a literature search focused on collecting, organising and analysing data related to cohesion, friction angle, and porosity of rocks and soils. The data collected were organised into a database containing more than 200 different lithotypes and analysed using different descriptive statistics. In addition, for the Italian territory, the collected information was used to associate geotechnical parameters to the lithological classes as identified by Bucci *et al*.^4^. These reclassified maps may provide researchers and stakeholders with a comprehensive dataset useful, in Italy, for different applications. In fact, several scientific fields can benefit from the product developed in this study, including regional/national-scale applications for (i) land management, (ii) urban planning, (iii) geological risk assessment, (iv) infrastructure design, and particularly (v) physically-based modelling for slope stability assessment^45,48–54^. It’s important to specify that the products of this study are not suitable for local-scale applications, as they are derived from small-scale cartography and are not capable and reliable to accurately represent local lithotype variations.

The manuscript is structured as follows: in the “Methods” section, the following are described: (i) the procedure for data searching through scientific journals; (ii) the criteria adopted to organise the collected information; (iii) the criteria selected to group the values into 18 lithological classes identified in Italy by Bucci *et al*.^4^; (iv) the analysis of the values of the geotechnical parameters collected for the different lithological classes, conducted using simple descriptive statistics.

In the “Data Records” section, readers will find the geospatial layers of the geotechnical characteristics of Italian lithotypes, available in vector format for GIS software, along with the percentile values and the coefficients of variation of geotechnical parameters such as cohesion, friction angle, and porosity.

Finally, in the “Technical Validation” section, the following are presented: i) the procedure for researching and organising data using grey literature; ii) a proposal for validating the parameters based on data collected from Italian grey literature.

## Methods

### Data collected from peer-reviewed journals

To collect information and compile a database on geotechnical parameters of rocks and soils, we adopted a systematic search of the scientific literature. To ensure the robustness and replicability of the procedure, we examined only peer-reviewed articles in Scholar and Scopus using key words and Boolean search criteria applied to the “title”, “abstract”, and “keywords” of the publications. Keywords included “geotechnical parameters of rocks”, “rock cohesion”, “shear strength parameters”, “friction angle”, “porosity”, and “rock laboratory tests”. The collected data were organised into a spreadsheet, where each record is a type of rock/soil as described in the original manuscripts. For each record, was recorded the following set of information:

(i) description of the manuscript (ID, authors, title, publication year, journal name, DOI);
(ii) approximate geographical location;
(iii) method used to acquire the parameter/s;
(iv) lithotype;
(v) values of the geotechnical parameters.

**Fig. 1:**
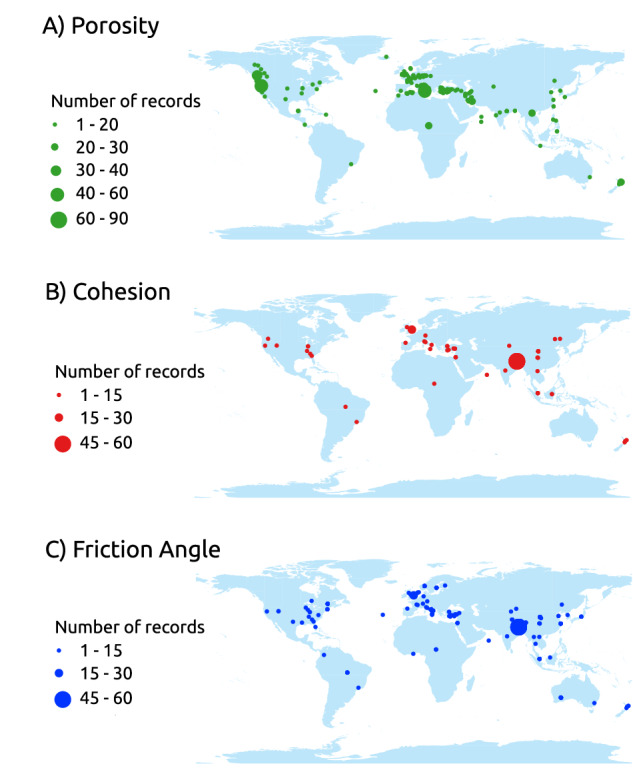
Spatial location of records listed in the database: (A) Porosity (1066 records), (B) Cohesion (542 records),and (C) Friction angle (732 records).

The search allowed to select a total of 102 scientific manuscripts published in 57 different journals. The top four journals (International Journal of Rock Mechanics and Mining Sciences, Engineering Geology, Journal of Rock Mechanics and Geotechnical Engineering and Scientific Reports) account for more than 35% of the total (Fig. 1). Among the papers, 56 provided data on cohesion, 59 on friction angle, and 49 on porosity.

**Fig. 2:**
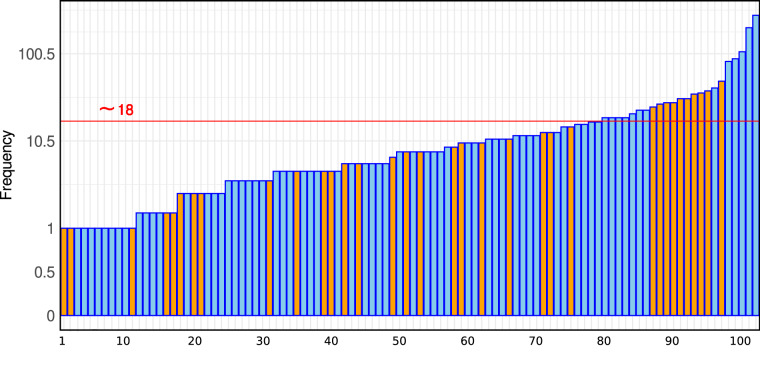
The graph shows the number of samples obtained from the 102 selected manuscripts, ranked by frequency. The orange bars represent the data collected from the four journals accounting for 35% of the total (International Journal of Rock Mechanics and Mining Sciences, Engineering Geology, Journal of Rock Mechanics and Geotechnical Engineering, and Scientific Reports).

The database includes information from more than 40 countries around the world (Fig. 2). The largest amount of information comes from Asia, accounting for 45,75% of records for cohesion, 45,50% for friction angle, and 31,90% for porosity. Conversely, there is a notable scarcity of information from Africa, Oceania, and South America.

In this article, the term ‘sample’ refers to the rock or soil specimen subjected to laboratory and/or field tests, as described by the authors. The term ‘record’, instead, refers to the specific set of data obtained for each sample. For each sample, it was possible to gather data (i.e., values) for one, two, or all three the geotechnical parameters namely cohesion, friction angle, and porosity.

The total number of samples derived from the 102 selected papers is 1775. The total number of records is 2338 and include 542 records with values of cohesion, 732 of friction angle, and 1066 of porosity. As shown in Table 1, 515 samples provide information for both cohesion and friction angle, and only 24 for the three parameters.

**Table 1:** The table shows the number of samples available for each combination of parameters and the total number of records.

Number of samples	Number of records for sample	Number of records
3	1 (cohesion)	3
193	1 (friction angle)	193
1040	1 (porosity)	1040
515	2 (cohesion and friction angle)	1030
24	3 (cohesion, friction angle and porosity)	72
**Total number of records**		**2338**

Figure 1 shows the number of samples obtained for each of the 102 selected papers. The average is 18 and the maximum (276) is derived from the article written by^55^.

**Fig. 3:**
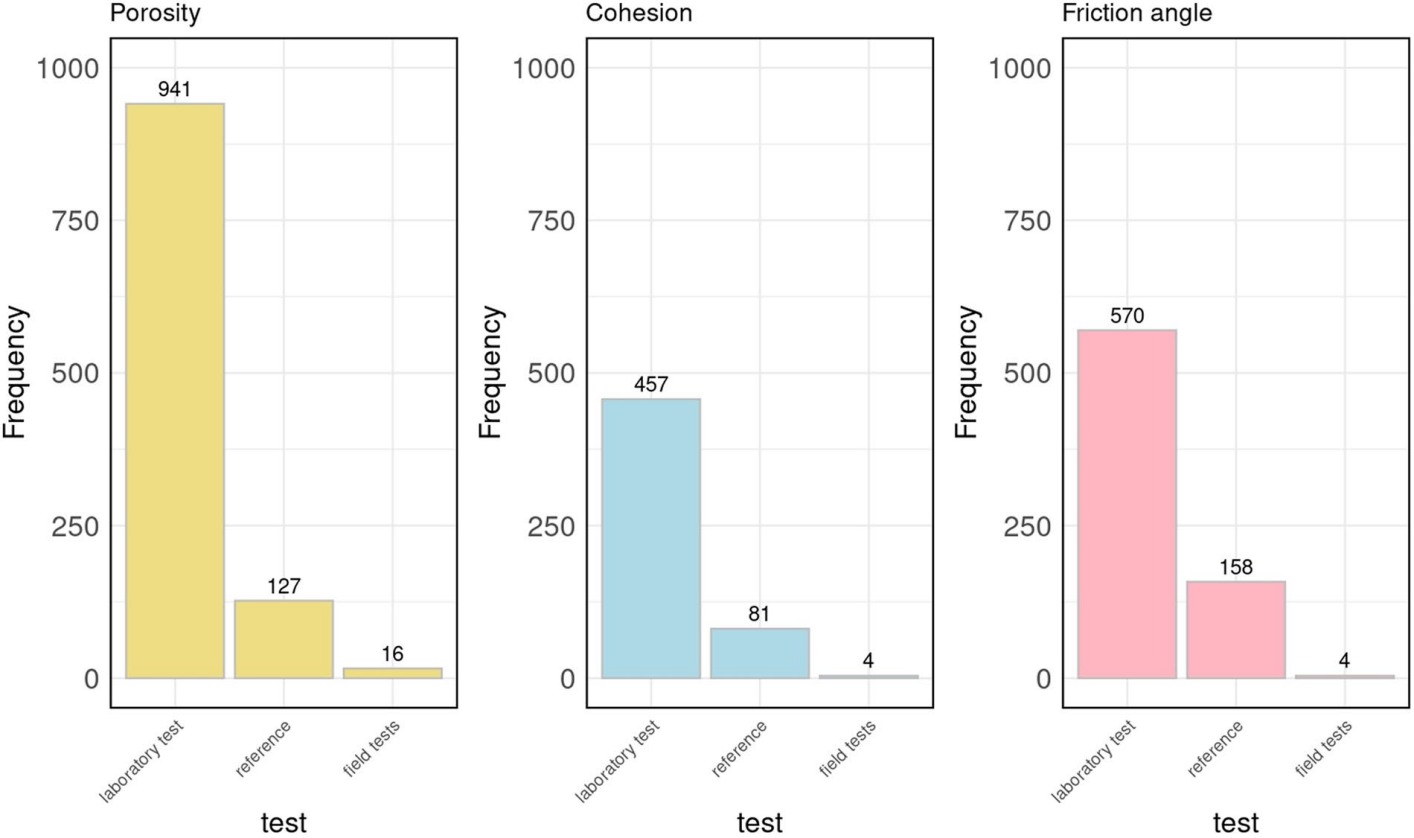
Methods used to obtain values of porosity, cohesion, and friction angle as described by the authors in the collected papers.

As described by the authors, values of geotechnical parameters were obtained using a considerable diversity of methods (Fig. 3) including laboratory tests, field tests and reliable sources previously published (we use the term “reference” to indicate data collected using this method). These sources include peer-reviewed articles, books, and other recognized academic sources.

The methods described for measuring cohesion and friction angle include both laboratory and field tests, which can be categorised as direct or indirect tests, as they evaluate the parameter directly or indirectly through equations. Laboratory tests include, among direct tests, triaxial tests^56^ and direct shear tests^57^, or both^58^; among indirect tests there are uniaxial tests^59^. Field tests include the Standard Penetration Test^60^.

We assume that the collected values of friction angle and cohesion pertain to drained conditions. In fact, the majority of the data were derived from standard (e.g., direct shear and triaxial tests) and non-conventional (e.g., shear creeping, etc) laboratory tests, or empirically-based relations from *in-situ* testing (e.g., NSPT-ϕ′ relationship), or conversions from other constitutive laws (e.g., from Hoek-Brown to Mohr-Coulomb^61,62^). These latter values of c′ and φ′ were directly collected from papers where the original authors had already performed the conversion from Hoek-Brown. All these parameters of cohesion, c′, and friction angle, ϕ′, are known as ‘effective’ and refer to the Mohr-Coulomb failure criterion. However, nearly 10% of the collected friction angles actually refer to the base friction angle of joints, ϕ′_b_, which is used to describe the non-linear shear behaviour of rock joints^63,64^. This parameter was estimated from laboratory tests, such as direct shear and tilt tests, as reported in the respective papers. We decided to consider also the ϕ′_b_ values as they describe a feature of the rock behaviour. However, these values are distributed across the 15 lithological classes, resulting in minimal impact on the boxplots.

The evaluation of porosity was conducted through laboratory tests including dry density measurements^65,66^, mercury porosimetry^67^, and estimates derived from measurements of P-wave velocity^44^.

Values of cohesion, friction angle and porosity are associated with 206 different lithotypes. This large number of lithotypes reflects both the characteristics of the materials and the choice of nomenclature adopted by the papers’ authors. For example, in the selected literature are mentioned several types of clays, including: (i) ‘sensitive clay’, i.e. clays deposited in marine depressions during the glacial era and later exposed to fresh water, resulting in reduced shear strength^68,69^; (ii) ‘marine clay’, i.e. characterised by a natural moisture content exceeding liquid limit^70^ and high compressibility, low strength, and low permeability^71–73^; (iii) ‘organic clay’, i.e exhibiting peculiar engineering properties such as low density, high compressibility, and large creep coefficients^74^.

During the data organisation, we noticed errors in the values attributed to the geotechnical parameters, probably caused by typing or transcription inaccuracies. In some cases, associating the value of the geotechnical parameter with a specific lithotype was questionable or highly doubtful. Additionally, some papers presented parameters related to contaminated lithotypes, where the presence of pollutants impacted the geotechnical values. In all cases, we adopted a rigorous approach, collecting only the values of the geotechnical parameters deemed correct and adequately associated with unaltered materials (rocks and soils).

Papers dealing with mixtures of lithotypes constituting artificial materials were discarded for the sake of consistency in the analyses, which needed to be exclusively focused on data derived from natural conditions.

Particular attention was paid to the homogeneity of units of measure. Cohesion values (expressed in KN/m² in some literature and in kPa in others) were converted to Megapascals (MPa). Similarly, porosity was not always expressed in percentage terms; in some cases, authors used alternative units or specific dimensional representations, creating an additional challenge in standardising the collected data. To further facilitate the analysis and ensure consistency within the project’s context, rock porosity was uniformly recorded in percentage.

It is important to specify that, regarding the parameters collected on stone materials, most of the information in the database comes from laboratory tests (Figs. 3, 6), particularly from triaxial tests. This implies that most of the samples analysed were taken *in situ* in good condition, without considering the degree of fracturing or alteration, unlike tests conducted directly in the field or those carried out to estimate ϕ’_b_.

### Characterization of the Italian lithotypes

We tried to use the dataset of geotechnical parameters to characterise the Italian lithotypes at national scale using the classes shown in the map published by Bucci *et al*.^4^. The map classifies different sedimentary, igneous and metamorphic rocks/terrains in 18 classes based on their lithological characterization.

The map by Bucci *et al*.^4^ does not include information on the values or ranges of geotechnical parameters to characterise the lithological classes, hence the information included in our database was used to fill this gap. The lithological map includes the following 18 lithological classes: Alluvial deposits (Al), Anthropogenic deposits (Ad), Beaches and coastal deposits (B), Carbonate rocks (Cr), Chaotic – mélange (Cm), Consolidated clastic rocks (Ccr), Evaporite (E), Glacial drift (Gd), Intrusive rocks (Ir), Lavas and basalts (lb), Marlstone (M), Mass wasting material (mw), Mixed sedimentary rocks (SM), Non-schistose metamorphic rocks (Nsr), Pyroclastic rocks (Pr), Schistose metamorphic rocks (Sr), Siliciclastic sedimentary rocks (Ssr), Unconsolidated clastic rock (Ucr). The 206 lithotypes in the database were associated with 15 (out of 18) classes, on an expert basis (see “Supplementary material”). The classes ‘Anthropogenic deposits’ (Ad), ‘Beaches and coastal deposits’ (B), and ‘Mass wasting material’ (mw) were excluded.

This association procedure highlighted that: (i) some lithological class of the Bucci *et al*.^4^ map may include several lithotypes listed in the database; and (ii) some lithotypes of the database can be associated with more than one lithological class.

The procedure used to associate the lithotypes of the database to the lithological class of the Bucci *et al*.^4^ map has shown different levels of complexity and uncertainty. For some lithological classes, which have modest internal variability and are geographically uniform, the association with lithotypes is clear and straightforward, such as in the case of volcanic or metamorphic rocks (i.e., Ir, Ib, Nsr, and Sr). Basalt, andesite, dacite, and riodacite were easily grouped in the “Lavas and basalt” class (Ib); similarly, schist, slate, and phyllite were reclassified in the “Schistose metamorphic rocks” class (Nsr).

For other lithological classes, which exhibit non-negligible internal variability, the relationships between classes and lithotypes were not unambiguous. For example, clays are common in the “Alluvial deposits” (Al), “Unconsolidated clastic rocks” (Ucr), and “Chaotic – mélange” (Cm). In addition, based on the geotechnical parameters considered and, where specified (e.g., “stiff over consolidated”), the clays were also associated with the lithological class of “Consolidated clastic rocks.” Similarly, sandstones are typical of the “Siliciclastic sedimentary rocks” (Ssr), but they are also common in the “Mixed sedimentary rocks” (SM) along with shales, clays, marls, and calcilutites. Regarding the marls, they have been associated with two lithological classes, “Marlstone” and “Mixed sedimentary rock,” based on the geotechnical parameters considered. The association of the lithotypes with the most heterogeneous lithological classes was managed according to a criterion of proportionality, and carefully considering the nature of the material (normally consolidated, overconsolidated, plastic, rigid, etc.), its nomenclature and geo-lithological information of the sampling sites (depositional environment, age, geostructural conditions, etc.). This reduced incorrect associations of lithotypes and lithological classes.

The geotechnical parameters associated to the different lithological classes of Bucci *et al*.^4^, were analysed using simple descriptive statistics (i.e., mean, median, and quartiles). Figure 6 shows in the left column, median values of cohesion, friction angle, and porosity computed for different lithotypes class and in the right column the quartile coefficient of variation (QCV^75^). QCV is a descriptive statistic which measures the data dispersion, defined as the ratio of the interquartile range to the sum of the two quartiles, and can be used to make comparisons within and between data sets.

**Fig. 4:**
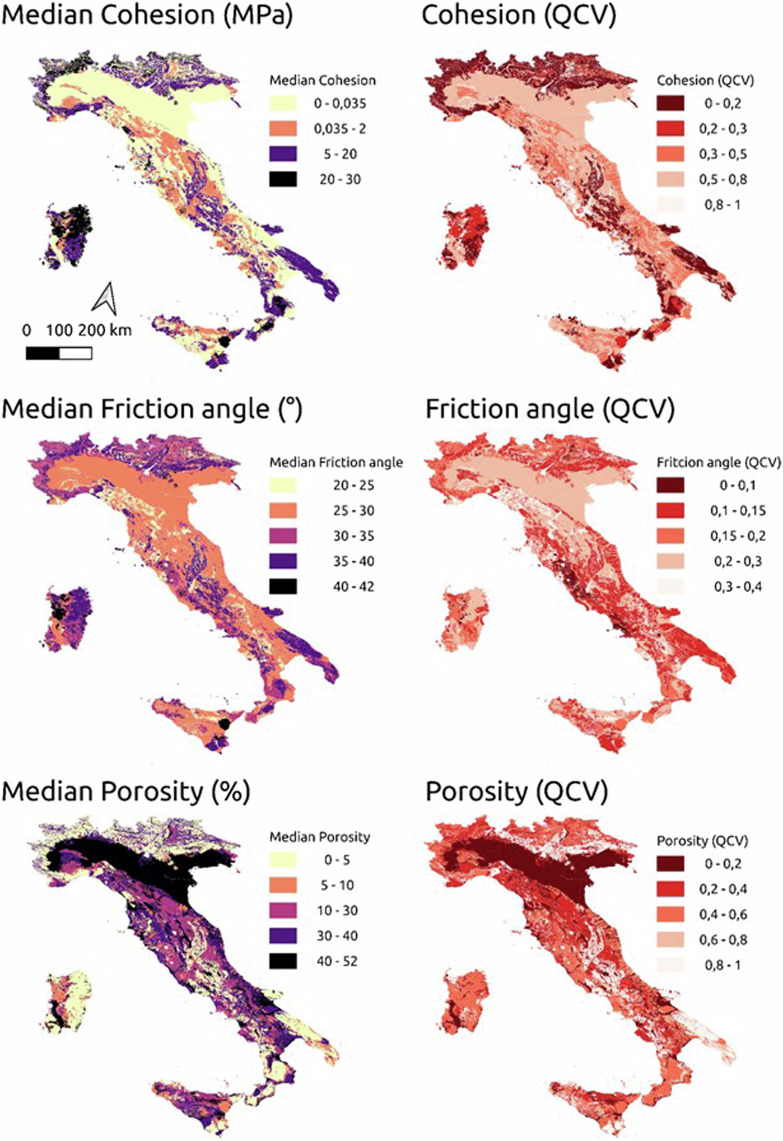
Median values of cohesion, friction angle, and porosity computed for different lithotypes (on the left) and in the coefficient of quartile variation (on the right). The values shown in the maps are not suitable for local-scale applications, as they are derived from small-scale cartography and are not capable and reliable to accurately represent local lithotype variations.

**Fig. 5:**
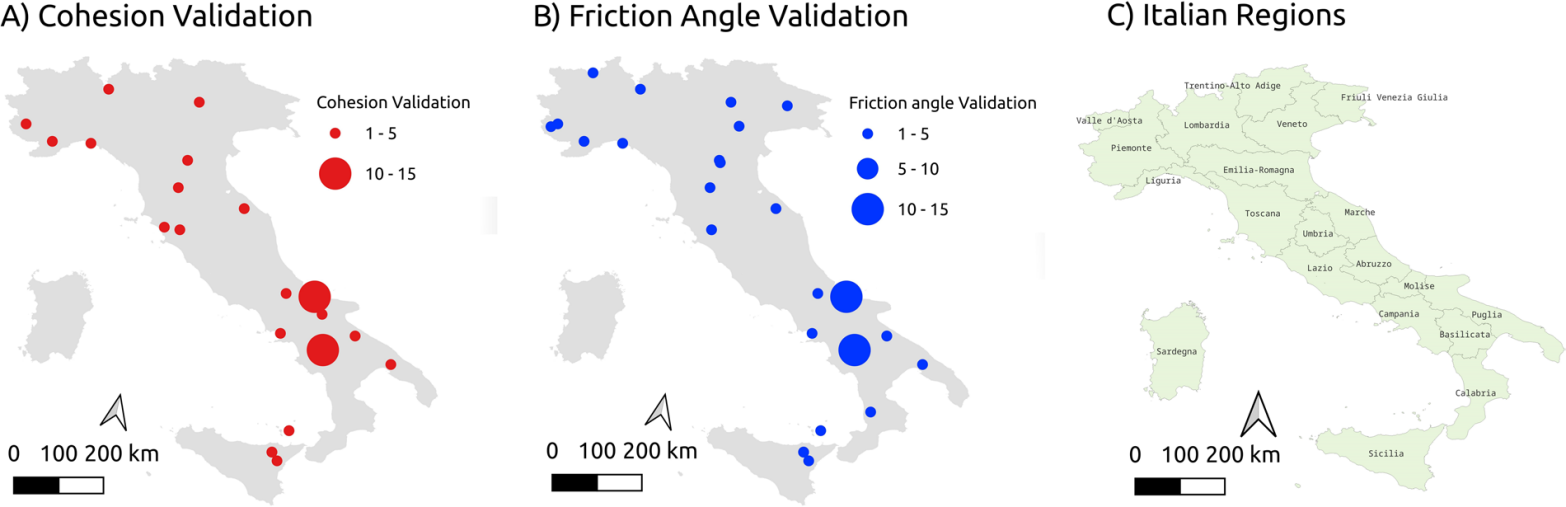
(**A**) Spatial Distribution of Records in the Validation Database for Cohesion. (**B**) Spatial Distribution of Records in the Validation Database for Friction Angle. (**C**) Map of Italy with region names. This map is provided to help readers locate the regions mentioned in the analysis.

Inspection of Fig. 4, reveals that the highest cohesion values (up to 30 MPa) are located in the Alpine arc, the plutonic complexes of the Sardinia region, the metamorphic rocks in Calabria, and the carbonate rocks in Puglia, central Italy, and southern Sicily (see Fig. 5 for the location of the Regions). Conversely, the lowest values (less than 0.035 MPa) are associated with rocks of sedimentary origin (Alluvial deposits, Chaotic – mélange, Mixed sedimentary rocks, Unconsolidated clastic rock, Evaporite). The greatest uncertainties are associated with the pyroclastic rocks in central Italy (0.8 < QCV < 1.0).

Median values of the friction angle follow a spatial pattern similar to the cohesion, although values have a narrow range (median values ranging from 20° to 42°) and are therefore more homogeneous at a national scale. The greatest uncertainties are associated with the Chaotic-melange, Marlstone and Mixed sedimentary rocks classes, although values are rather small (0.3 < QCV < 0.4).

Median porosity value is maximum in the alluvial deposit and is minimum for the plutonic or metamorphic rocks (ranging from 0 to 5%). The uncertainty is very low for the alluvial deposits (0 < QCV < 0.2) and high for carbonate rocks (0.8 < QCV < 1.0), reflecting the duality between primary and secondary porosity of these lithological classes. The presence of two distinct types of porosity, each with vastly different physical properties, introduces substantial variability in the porous structure of carbonate rocks. This variability makes it challenging to accurately and consistently estimate their total porosity. The resulting high level of uncertainty is largely driven by the complex interplay of factors such as local geological history, the nature of fracturing, and the varying intensity of dissolution processes, all of which can differ significantly across different spatial scales. The data related to Fig. 4 are available on Figshare^76^.

## Data Records

The geospatial layers describing the geotechnical characteristics of the Italian lithotypes, and shown in Fig. 4, are available at [10.6084/m9.figshare.26326537.v1] in Vector format (filename: Geotech_Italy.gpkg^76^) compliant with GIS software, such as QGIS, ArcGIS, or others. In the attribute table of the maps, the 25th percentile, median and 75th percentile of the values of the three geotechnical parameters (i.e., cohesion, friction angle, and porosity) are reported, with the following field names: “cohesion_25percentile_Mpa”, “cohesion_75percentile_Mpa”, “cohesion_median_MPa”, “friction_angle_25percentile_degrees”, “friction_angle_75percentile_degrees”,“friction_angle_median_degrees”, “porosity_25percentile_percentage”, “porosity_75percentile_percentage”, “porosity_median_percentage”. In addition, for each geotechnical parameter, the quartile coefficient of variation (QCV) is listed in the attribute table: “cohesion_QCV”, “Friction_angle_QCV”, “Porosity_QCV”.

The values shown in the maps are not suitable for local-scale applications, as they are derived from small-scale cartography and are not capable and reliable to accurately represent local lithotype variations.

## Technical Validation

### Data collected from the grey-literature

To validate the expert-based associations between lithotypes and the lithological classes of Bucci *et al*.^4^, we collected additional data and stored them in a “validation database”, independent from the main one. The validation database was compiled exclusively from grey literature, which includes sources not typically subject to peer review or widely published. These sources encompass unpublished documents, academic theses, conference proceedings from research institutions, as well as design documents, technical reports from private companies, books, and relevant web pages. The data were collected only from Italian sources. Figure 5 shows the spatial distribution of the data.

Data collection for the validation set was performed following the same approach used for the main database. For each sample, representing a particular type of rock or soil, all relevant information such as ID, authors, report title, geographic location, and data acquisition methodology were recorded in a spreadsheet.

**Fig. 6:**
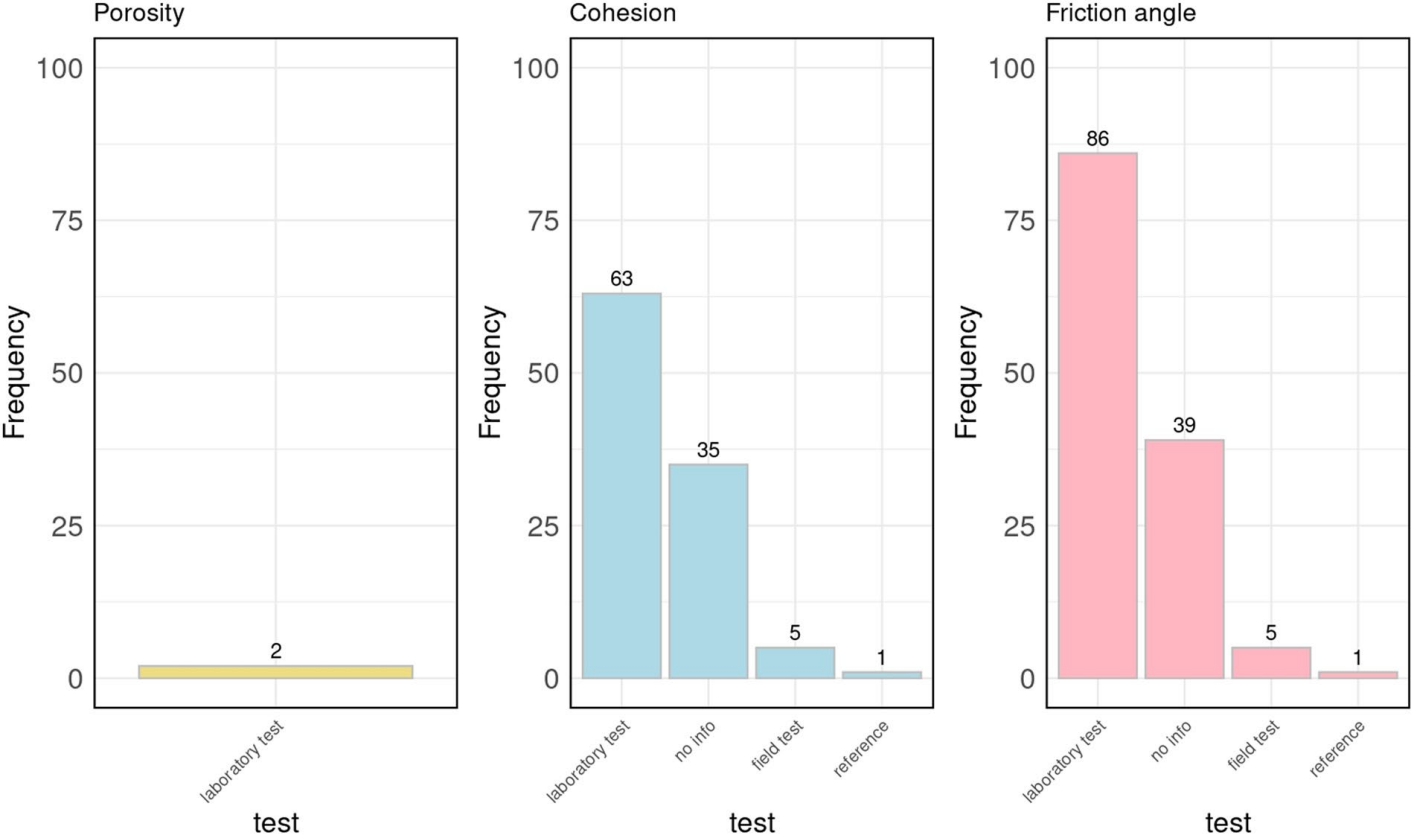
Methods used to obtain values of porosity, cohesion, and friction angle as described by the authors in the grey literature.

In this case, 32 reports were collected, of which 24 provide data on cohesion, 31 on friction angle, and only one on porosity. The total number of samples is 148, and the total number of records is 237, 104 of which on cohesion, 131 on friction angle, and 2 on porosity. As shown in Table 2, information on both cohesion and friction angle is available for 85 samples, and information on all three parameters is available for only 2 samples. Regarding the data acquisition methods (Fig. 6) both laboratory tests and field tests were employed to obtain values of cohesion and friction angle. Laboratory tests included triaxial tests, direct shear tests, or both, while the Standard Penetration Test was used for field tests.

**Table 2:** The table shows the number of samples available for each combination of parameters and the total number of records collected in Italy using the grey literature.

Number of samples	Number of records for sample	Number of records
17	1 (cohesion)	17
44	1 (friction angle)	44
85	2 (cohesion and friction angle)	170
2	3 (cohesion, friction angle and porosity)	6
**Total number of records**		**237**

### Validation

**Fig. 7:**
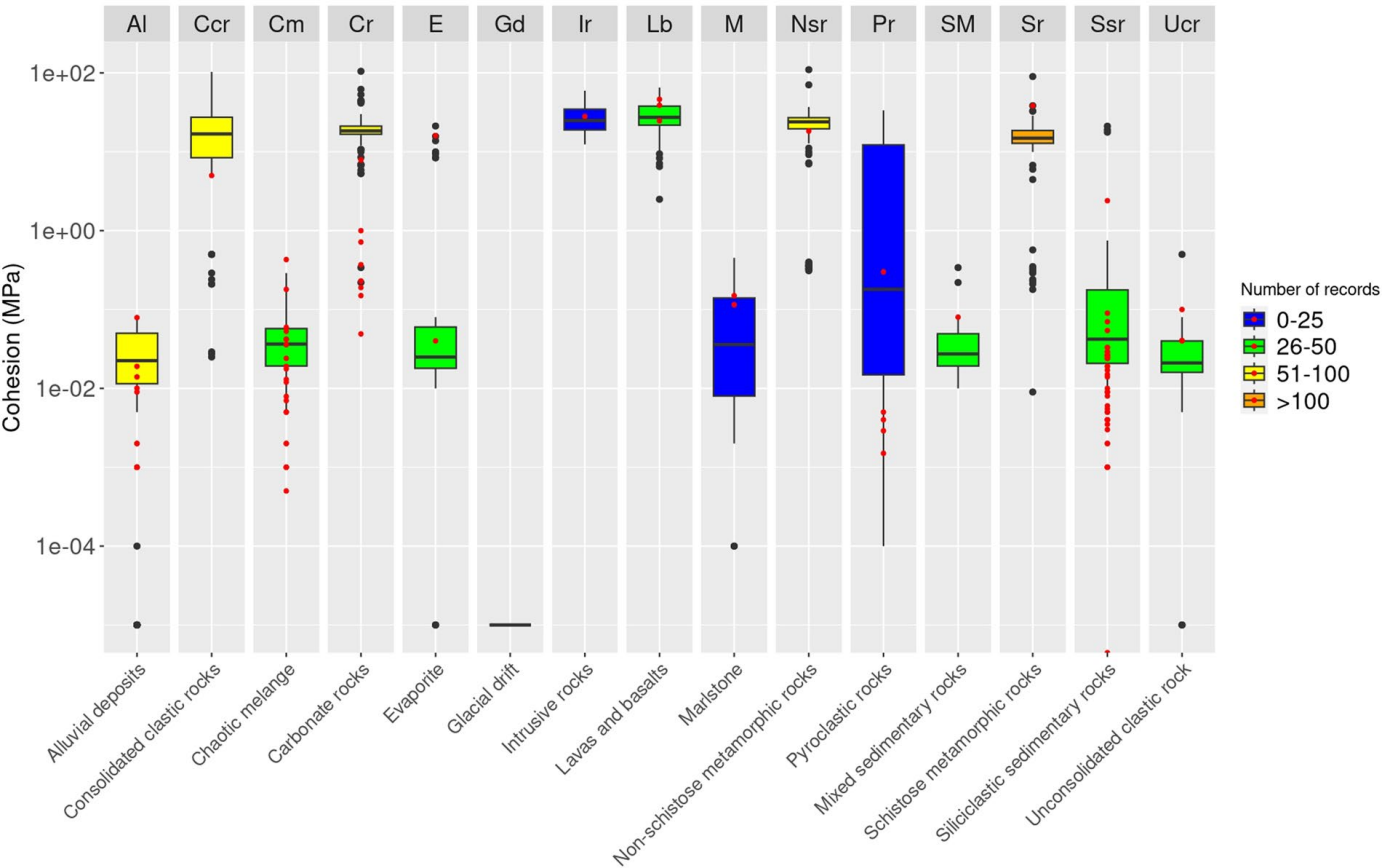
Boxplots for values of cohesion classified in15 lithological classes Bucci *et al*.^4^. Red dots represent data collected from the Italian grey-literature and used as validation.

**Fig. 8:**
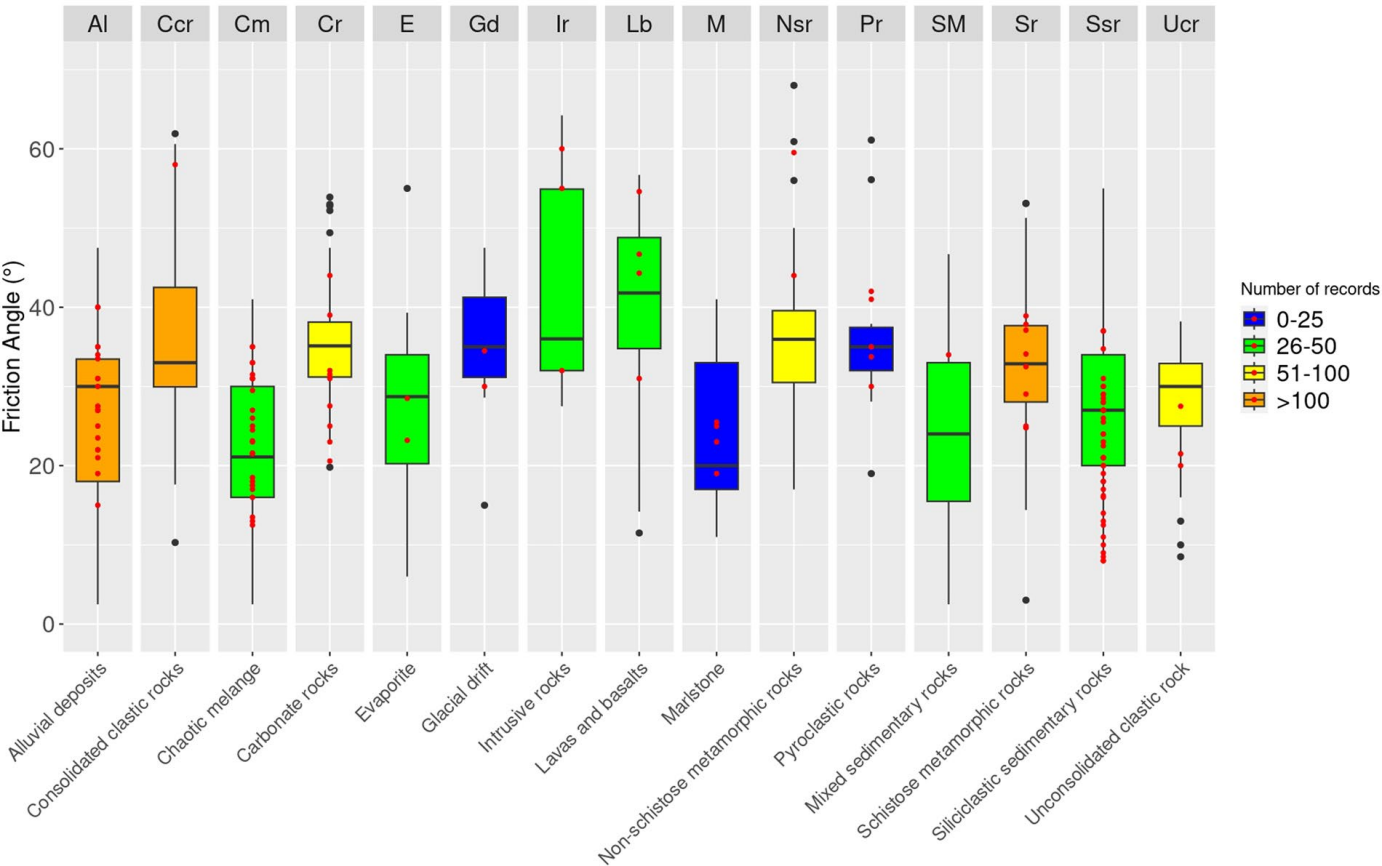
Boxplots for values of friction angle classified in15 lithological classes Bucci *et al*.^4^. Red dots represent data collected from the Italian grey-literature and used as validation.

**Fig. 9:**
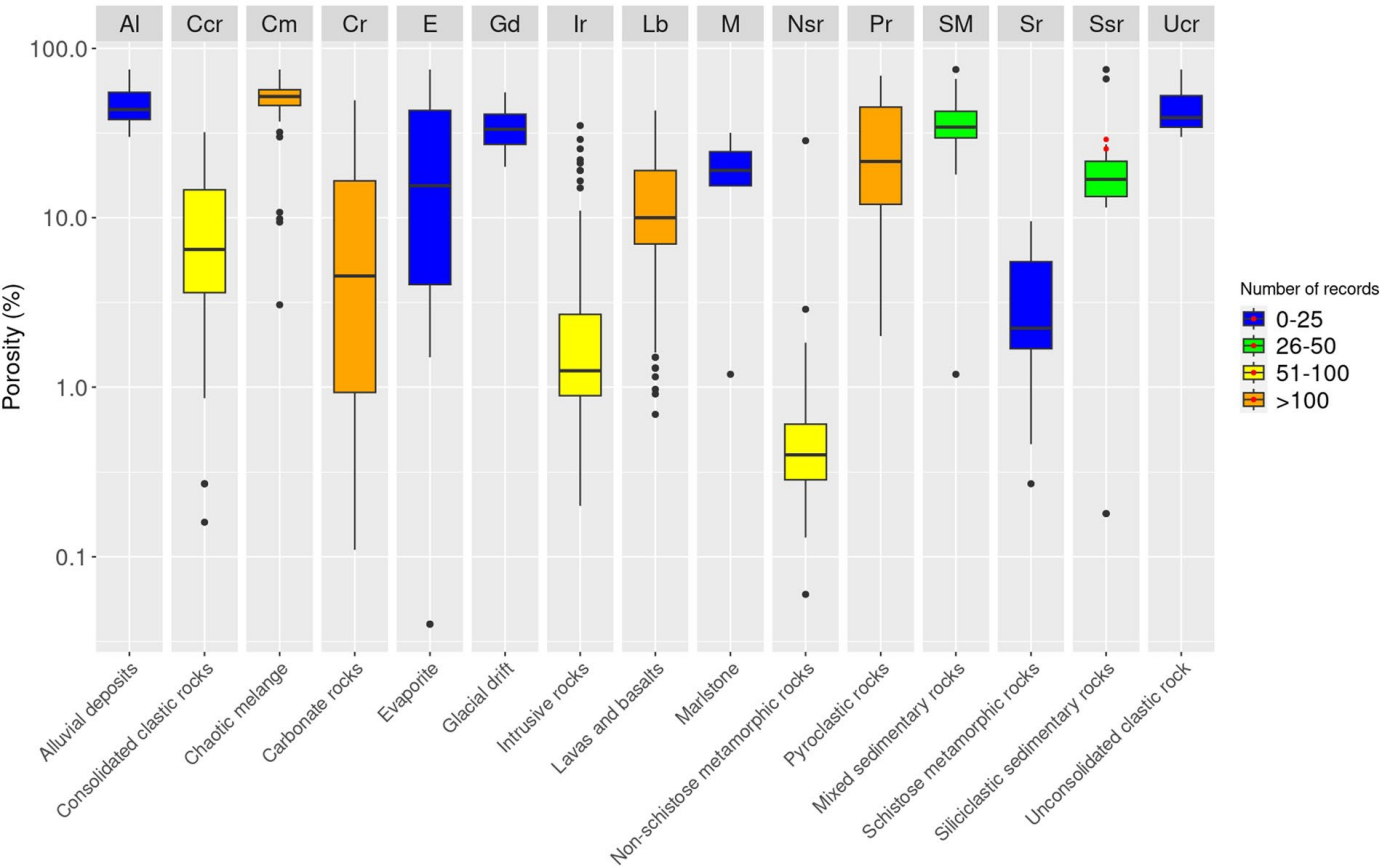
Boxplots for values of porosity classified in 15 lithological classes Bucci *et al*.^4^. Red dots represent data collected from the Italian grey-literature and used as validation.

To compare and evaluate the distribution of the parameters associated with the lithological classes as defined by Bucci *et al*.^4^, we prepared boxplots of the cohesion, friction angle, and porosity (Figs. 7, 8, 9). Boxplots for the cohesion (Fig. 7) were prepared for 12 lithological classes where more than 25 data points were available. Inspection of the graph, highlights the high cohesion values of volcanic, metamorphic, and carbonate lithological classes (Sr, Nsr, lb, Ir, Cr). Consolidated clastic rocks (Ccr) also show comparable cohesion values (~10^1 MPa) but exhibit a greater variability. Cohesion values for other lithological classes are generally around three orders of magnitude lower (~10^-2 MPa) and encompass both soil-like materials (Al, Ucr) and rock-like materials (Cm, E, M, SM, Ssr). Among these, Marlstone (M) and Siliciclastic Sedimentary Rocks (Ssr) exhibit greater variability. Surprisingly, pyroclastic rocks (Pr) exhibit a variability range spanning three orders of magnitude. We maintain this is due to the presence of pyroclastic terms ranging from unconsolidated, loose materials to lapilli tuffs within the same lithological class.

This is because, in the lithological classification by Bucci *et al*.^4^, the pyroclastic rock class includes both tuffs and volcanic breccias, which can exhibit high cohesion due to their greater compaction and cementation, as well as materials such as ash, scoria, and pumice, which tend to have very low cohesion due to their more porous and less consolidated structure. This wide variability in cohesion makes it difficult to generalise the mechanical properties of pyroclastic rocks, as their behaviour can vary significantly depending on their composition and degree of consolidation.

In the same plot (Fig. 7), data points from the validation dataset are plotted as red points. The overlap of boxplots and validation data points highlights: (i) a good correspondence for the Lb, Ir, Nsr, M classes (although there are few validation data points available), (ii) an acceptable similarity in the Al, Cm, Ssr classes, (iii) an overestimation for the Cr, Pr, Ccr classes, and an underestimation for the E, SM, Sr, Ucr classes. No validation data were identified for Gd.

The number of data points used to analyse the friction angle values (Fig. 8) is similar to that used for cohesion, with most classes represented by over 25 data points. The graph reveals three main clusters: the first includes lithologies such as Ir, Lb, Gd, and Ccr, where values can exceed 40° and rarely drop below 30°. The second group, with values ranging between 28° and 40°, includes Cr, Nsr, Sr, and Pr. The third group, with friction angles often between 16° and 35°, comprises Al, Cm, E, M, SM, Ssr, and Ucr. Validation data (red point in Fig. 8) are sufficiently consistent with the values in all lithological classes except for a slight overestimation for Ssr and Ucr and underestimation for Ccr, SM, and Nsr classes (however, with few validation data).

Figure 9 shows boxplots related to porosity data. The number of data points is higher than cohesion and friction angle, clustered in few lithological classes. The boxplots reveal two groups of lithological classes based on porosity. One group includes lithoid rocks characterised by low porosity (i.e., Cr, Ir, Lb, Nsr, Sr, and Ccr). The other includes rocks with higher porosity and represents lithological classes with significant grain size variability or the presence of stratifications/fractures that determine an important secondary porosity (i.e., Al, Gd, Cm, Ucr, Pr, E, M, SM, and Ssr). Unfortunately for porosity, it was not possible to acquire a statistically significant number of validation data points.

Among the lithological classes identified by Bucci *et al*.^4^, some are characterised by high granulometric and mechanical heterogeneity. The classes Cm, CCr, and Al can locally exhibit either markedly frictional characteristics (with stratifications of sandy and/or coarse-grained material, sometimes even with rock-like behaviour) or cohesive characteristics (with predominant clayey or silty layers in stratified or chaotic settings). To investigate whether the values of geotechnical parameters characteristic of these lithological classes can be significantly influenced by these local alterations or variations, two alternative hypotheses of lithotype association were formulated to simulate the different nature of the Cm, CCr, and Al classes: (i) in the first case, only gravelly-sandy lithotypes (coarse member) were considered, and (ii) in the second case, only clayey-silty lithotypes (fine member).

**Fig. 10:**
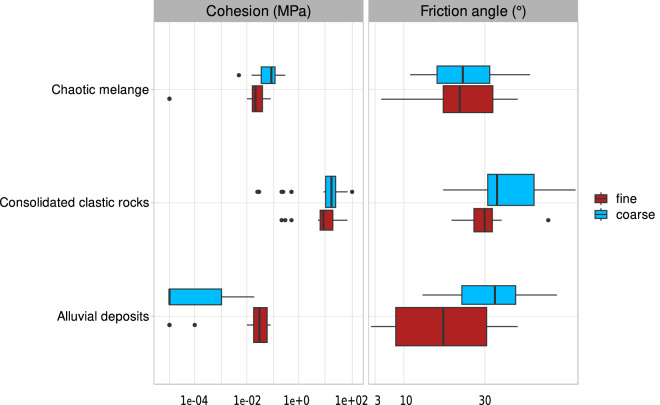
Box plots of cohesion and friction angle for the fine (red) and the coarse (light blue) component of three lithological classes.

The distributions of cohesion and friction angle values are shown in the boxplots in Fig. 10, where the coarse and fine members are represented, respectively, with blue and red boxplots. As expected, the friction angle is significantly higher for the coarse members, except for the Cm class, where the friction angle shows a substantially identical distribution for both members. For this latter class, such behaviour can be explained by the high degree of tectonization and alteration of the materials, which impacts its mechanical properties and was considered by the authors in the decision-making process of associating lithotypes with the class. The Cm class also shows a very different behaviour from the other classes concerning cohesion, which is significantly higher for the coarse members compared to the fine ones. This result can also be attributed to the presence of markedly lithoid materials (rocks, hence very cohesive) within this lithological class, as noted by the authors.

For the Al class, significant differences are also observed between the cohesion values for the coarse and fine members. However, as expected, the latter presents a distribution of values well above the former and similar to that of the Cm class, as both are characterised by an abundance of clays. The distributions of cohesion values for the two members of the CCr class are very similar, with a slight (though not significant) prevalence for the coarse materials, which can be attributed to the presence of lithoid materials and cementation between clasts.

This work is an attempt to associate values of some geotechnical parameters to lithological classes. In particular, we have exploited data/information derived from a literature search to characterise the lithological classes proposed by Bucci *et al*.^4^ for the Italian territory. However, it is crucial to emphasise that the reclassified maps and the associated data should be used with extreme caution. These maps are intended for broad, national/regional-scale analyses and are not suitable for local-scale applications, as they cannot capture the inherent variability of geotechnical parameters at finer scales. Therefore, users should avoid applying these datasets directly to detailed geotechnical design or local land management as the values shown in the maps are not suitable for local-scale applications, as they are derived from small-scale cartography and are not capable and reliable to accurately represent local lithotype variationsthe application at local scale may lead to inaccurate/erroneous assessments and potential legal implications. Proper considerations on the scale and limitations of the data are essential to ensure their appropriate and correct use and application.

## Supplementary information

Association of Lithotypes with Italian Lithological Classes by Bucci et al. (2022)

## Data Availability

No specific custom code was used in the execution of this work.
