# Supplementary material for: A dataset of geotechnical parameters based on international literature to characterise lithotypes in Italy
Source: Sci Data. 2024 Dec 18;11:1371. doi: 10.1038/s41597-024-04095-1 (PMC11655648; doi:10.1038/s41597-024-04095-1)
Supplement: Supplementary file 1 — Association of Lithotypes with Italian Lithological Classes by Bucci et al. (2022) [file 41597_2024_4095_MOESM1_ESM.doc]

| **L** | **LC** |  | **L** | **LC** |  | **L** | **LC** |
| --- | --- | --- | --- | --- | --- | --- | --- |
| alabaster gypsum-anhydrite | E |  | clay (montmorillonite) | Al |  | debris flows | Cm |
| alluvial clayey silt | Al |  | clay (montmorillonite) | Cm |  | diorite | Ir |
| alluvial deposit | Al |  | clay (montmorillonite) | E |  | dol. limestone | Cr |
| altered andesite lava | Lb |  | clay (montmorillonite) | SM |  | dolerite | Ir |
| amarelo pais granite | Ir |  | clay (montmorillonite) | Ssr |  | dolomite | Cr |
| amphibolite | Nsr |  | clay (montmorillonite) | Ucr |  | dolomitic limestone | Cr |
| andalusite garnet hornfels | Nsr |  | claynstone bedding | Ccr |  | dolostone | Cr |
| andesite | Lb |  | coal | Ssr |  | evaporite | E |
| andesite lava | Lb |  | coarse marble | Nsr |  | fine sand | Ucr |
| anhydrite | E |  | coarse-grained granite | Ir |  | fine sandstone | Ccr |
| arenaceous marl | M |  | cohesive soil (clay, silt, sand) | Ccr |  | fine-grained granite | Ir |
| argillaceous sandstone | Ccr |  | soft plastic clay | Al |  | firm marine clay | Ccr |
| argillite | Ccr |  | completely altered ash tuff | Pr |  | firm nc silty clay | Al |
| soft estuarine clay | Al |  | conglomerate | Ccr |  | firm organic clay | Ccr |
| basalt | Lb |  | dacite | Lb |  | firm silty clay | Ccr |
| basalt lava | Lb |  | carbonate | Cr |  | fissured london clay | Al |
| basaltic-andesite | Lb |  | chalk | E |  | nc phosphatic clay | Al |
| biotite chlorite schist | Sr |  | chaotic complex | Cm |  | flow | Cm |
| blanco mera granite | Ir |  | clay | Al |  | flysch | Ssr |
| block-and-ash flow | Pr |  | clay | Cm |  | gabbro | Ir |
| breccia | Cm |  | clay | E |  | glacial clay | Gd |
| breccia | Cm |  | clay | SM |  | glacial till | Gd |
| bunt sandstone | Ccr |  | clay | Ssr |  | glaciomarine clay | Gd |
| calcarenites | Cr |  | clay | Ucr |  | glaciomarine silt | Gd |
| calcarenites | SM |  | clay (illite/kaolinite) | Al |  | gneiss | Nsr |
| calcareous cooper marl | SM |  | clay (illite/kaolinite) | Cm |  | gneiss | Sr |
| calcareous tuff | SM |  | clay (illite/kaolinite) | E |  | gosford sandstone | Ccr |
| calcite–quartz conglomerate | Ssr |  | clay (illite/kaolinite) | SM |  | granite | Ir |
| calcite–quartz sandstone | Ssr |  | clay (illite/kaolinite) | Ssr |  | granite gneiss | Sr |
| calcite sandstone | Ssr |  | clay (illite/kaolinite) | Ucr |  | granite basalt | Lb |
| granitoid | Ir |  | migmatite | Nsr |  | sand with clay binder | Al |
| granodiorite | Ir |  | mikaschist | Sr |  | sand with clay binder | Ucr |
| gravel | Al |  | monzogranite | Ir |  | sandstones | Ccr |
| gravel | Gd |  | mudstone | Ccr |  | sandy mudstone | Ccr |
| gravel | Ucr |  | mudstone | Ucr |  | schist | Sr |
| green shale | Sr |  | norite | Ir |  | sedimentary rock | Ucr |
| gypsum | E |  | oc fissured gault clay | Ucr |  | sensitive clay | Al |
| halite | Cr |  | oc organic silt | Ccr |  | sensitive leda clay | Al |
| hard oc clay | Ccr |  | oc sensitive clay | Ccr |  | sensitive marine clay | Ucr |
| hololeucogranite | Ir |  | oc stiff clay | Ccr |  | sensitive nc clay | Al |
| hornfels | Nsr |  | offshore marine clay | Ucr |  | serpentinite | Sr |
| ignimbrite | Pr |  | onyx | Ssr |  | serpentinized olivine | Sr |
| illite | Cm |  | organic clay | Al |  | shale | Sr |
| impact melt rock | Ir |  | pervasively oxidised dacite | Lb |  | shale bedding | Sr |
| inorganic clay | Al |  | phyllite | Sr |  | shell limestone | Cr |
| ironstone | Ccr |  | piroclastiti | Pr |  | sillimanite garnet hornfels | Nsr |
| Kaolinite | Cm |  | porous sandstone | Ccr |  | silt | Al |
| Kirthai granite gneiss | Sr |  | porphiry | Ir |  | silt | Cm |
| laminated clay | Ucr |  | porphyry gypsum-anhydrite | E |  | silt | SM |
| lava | Lb |  | pyritiferous slate | Sr |  | silt-clay-gravel | Gd |
| lava+breccia | Lb |  | pyroclastic | Pr |  | silt-clay-gravel | Al |
| limestone | Cr |  | quartz diorite | Ir |  | siltstone | Ccr |
| lithic graywacke | Ssr |  | quartz mica schist | Sr |  | siltstone bedding | Ccr |
| loam | Al |  | quartz monzonite | Ir |  | silty clay | Al |
| lower friable sandstone | Cm |  | quartz sand | Al |  | silty clay till/clayey silt till | Al |
| marble | Nsr |  | quartz sandstone | Ssr |  | silty sandstone | Ssr |
| marine clay | Ucr |  | quartzite | Nsr |  | slate | Sr |
| marl | M |  | quartzite mica schist | Sr |  | slide | Cm |
| marl | SM |  | quartzwacke | Ssr |  | slumps | Cm |
| marl clay | M |  | rajnagar marble | Nsr |  | soft alluvial atchafalaya clay | Al |
| marlite | M |  | rhyolite | Lb |  | soft alluvial clay | Al |
| marlstone | Ccr |  | rhyolite | Lb |  | soft bay mud | Ucr |
| marlstone limestone | Cr |  | rock mass | Cm |  | soft carbonate clay | Al |
| massive blocks | Pr |  | rock salt | E |  | soft clayey silt | Al |
| massive sandstone | Cm |  | sand | Al |  | soft deltaic clay | Al |
| medium marble | Nsr |  | sand | Ucr |  | soft empire clay | Cm |
| metargillite | Ccr |  | sand with a lot of fine particles | Al |  | soft estuarine clay | Al |
| micaschist | Sr |  | sand with a lot of fine particles | Cm |  | soft glacial clay | Gd |
| soft glacial clay | Gd |  | sand with a lot of fine particles | Ucr |  | soft inorganic clay | Ucr |
| soft lacustrine clay | Al |  | stiff clay | Ccr |  | varved sediments | Ccr |
| soft leda clay | Al |  | stiff oc clay | Ccr |  | vesicular blocks s with small voids | Pr |
| soft marine clay | Ucr |  | stiff oc deltaic clay | Ccr |  | vesicular blocks with large voids | Pr |
| soft offshore clay | Ucr |  | suevite | Ir |  | volcanic bomb | Pr |
| soft organic clay | Al |  | sweden soft inorganic clay | Al |  | vulcanic tuff | Pr |
| soft sensitive clay | Al |  | syenite | Ir |  | weak volcaniclastic sediment | Pr |
| soft silty clay | Al |  | tonalite | Ir |  | Westerley granite | Ir |
| stanstead granite | Ir |  | trachyte | Lb |  |  |  |
| staurolite andalusite schist | Sr |  | traki-andesite | Lb |  |  |  |
| stiff calcareous clay | Al |  | travertine | Cr |  |  |  |
| stiff carbonate sandy clay | Ccr |  | tuff | Pr |  |  |  |

Where:

L=Lithotype

LC= Litological classes

Note:

The table has been created in alphabetical order; if it is modified, this order will no longer be preserved.
